# Ferroptosis-associated candidate genes in aneurysmal subarachnoid hemorrhage: evidence from human peripheral blood and mouse brain

**DOI:** 10.3389/fneur.2026.1823039

**Published:** 2026-10-05

**Authors:** Jia Li, Cong Wang, Hongwen Li, Yafan Liu, Chunchao Cheng, Zilin Zhao, Guifang Jiang, Xiaopeng Cui, Ben Chat Fong Ng, Yan Zhao, Zhuolin Wu, Xinyu Yang

**Affiliations:** 1 Department of Neurosurgery, Tianjin Medical University General Hospital, Tianjin, China; 2 Neurosurgery Third Department, Baoding No.1 Central Hospital, Baoding, Hebei, China; 3 School of Pharmaceutical Science, University of South China, Hengyang, China; 4 Department of Neurosurgery, Tianjin Fifth Central Hospital, Tianjin, China; 5 Department of Neurosurgery, Queen Elizabeth Hospital, Hong Kong, Hong Kong SAR, China

**Keywords:** bioinformatics, ferroptosis-associated genes, immune infiltration, aneurysmal subarachnoid hemorrhage, weighted gene co-expression network analysis

## Abstract

**Background:**

Aneurysmal subarachnoid hemorrhage (aSAH) has high morbidity and mortality. Ferroptosis may contribute to aSAH-related brain injury, but its peripheral and brain-associated signatures remain unclear.

**Methods:**

Human peripheral-blood dataset GSE36791 (43 aSAH, 18 controls) was analyzed using limma, FerrDb intersection, WGCNA, GO/KEGG enrichment, and PPI-based prioritization. qPCR validation was performed in peripheral blood from 10 aSAH patients and 10 non-SAH controls; aSAH samples were collected within 24 h after rupture. Mean ages were 60.2 ± 7.7 and 63.6 ± 8.9 years, with 5 and 4 males, respectively. Brain-level validation used GSE79416 mouse cortex and OMIX006611 mouse SAH single-cell RNA-seq data. Male C57BL/6 J mice (8 weeks, 20–22 g) underwent cisterna magna autologous blood injection; brain tissues were collected 1 day after SAH (n = 6/group). Immune infiltration and molecular docking were also performed.

**Results:**

Among 2,645 DEGs, 35 were ferroptosis-related. MAPK3, STAT3, G6PD, and PGD were prioritized as ferroptosis-associated candidate genes and were upregulated in human aSAH peripheral blood, mouse SAH cortex, and the clinical qPCR cohort. Single-cell analysis showed expression of the corresponding mouse genes across annotated brain cell types, especially immune/glial populations. In mouse SAH brain tissue, MAPK3/ERK1, STAT3, PGD, and G6PD protein levels increased, p-STAT3/STAT3 ratio was elevated, MDA content increased, and GPX4 activity decreased. Immune profiling showed increased neutrophils, mast cells, and activated dendritic cells. Ganoderic acid A was prioritized as a preliminary in silico candidate.

**Conclusion:**

MAPK3, STAT3, G6PD, and PGD are ferroptosis-associated candidate genes in aSAH, supported by human peripheral-blood, mouse cortex, single-cell, and mouse brain validation. Ganoderic acid A remains an exploratory in silico candidate requiring further functional validation.

## Introduction

Intracranial aneurysm rupture, resulting in aneurysmal subarachnoid hemorrhage (aSAH), is a fatal and highly disabling subtype of stroke (1). Within the first 72 h after aSAH onset, patients experience intricate pathophysiological processes, such as blood–brain barrier disruption, cerebral edema, and other modifications collectively known as early brain injury (EBI) (2). The exact molecular mechanisms of EBI-aSAH are currently being elucidated, but it is widely recognized that increased oxidative stress, blood–brain barrier disruption, reduced cerebrovascular flow, neuroinflammation, and cell death in the brain lead to secondary brain injury, and many studies have implicated ferroptosis in these processes and provided new ideas for the treatment of aSAH (2, 3). With the increase of subarachnoid hemorrhage, the rapidly increasing concentration of iron ions will become an inducer of lipid peroxidation during ferroptosis, and act as a catalyst to participate in the Fenton reaction, which generates a variety of free radicals, such as ROS. Then, membrane phospholipids containing polyunsaturated fatty acids are directly oxidized to lipid hydroperoxides under the action of lipoxygenase (LOX), and the overaccumulation of reactive oxygen species and lipid peroxides ultimately leads to cellular iron degeneration (4–6).

We employed data mining and data analysis methods to identify DEGs in blood samples from individuals with aSAH and healthy controls. We also used CIBERSORT and ssGSEA to identify immune infiltration and used methods such as weighted correlation network analysis (WGCNA) to elucidate the most relevant genes. Finally, we validated the results based on the GEO dataset using real-time quantitative fluorescence (qPCR) in the cohort. Our findings provide new key genes for ferroptosis associated with the progression of aSAH, which may aid in individualized diagnosis and treatment. Specifically, we analyzed differential gene expression in peripheral blood transcriptomics to explore systemic molecular changes following aSAH.

## Methods

### Dataset source

Gene expression profiling data (GSE36791, GSE79416, OMIX006611) obtained from the Gene Expression Omnibus (GEO) database and the National Genomics Data Center (NGDC) (7, 8). GSE36791 includes peripheral blood samples from 18 control groups, 43 ruptured aneurysm (aSAH) groups (9). The genes related to ferroptosis were obtained from FerrDb database (http://www.zhounan.org/ferrdb/legacy/index.html) (10). It presents 288 genes linked to ferroptosis, comprising of 108 drivers, 69 repressors, and 111 markers. We have handpicked a total of 214 genes entailed with *Homo sapiens*. GSE79416 was used to validate the expression of candidate hub genes in mouse cerebral cortex after SAH. The mouse orthologs corresponding to human MAPK3, STAT3, G6PD, and PGD, namely Mapk3, Stat3, G6pdx, and Pgd, were extracted for analysis (11). OMIX006611 was used to determine the cell-type-specific expression patterns of the candidate hub genes after SAH (12). GSE16561 and GSE89866 were used as ischemic stroke and traumatic brain injury comparison datasets, respectively, to provide comparative evidence (13, 14). The data processing flowchart is shown in Figure 1.

**Figure 1:**
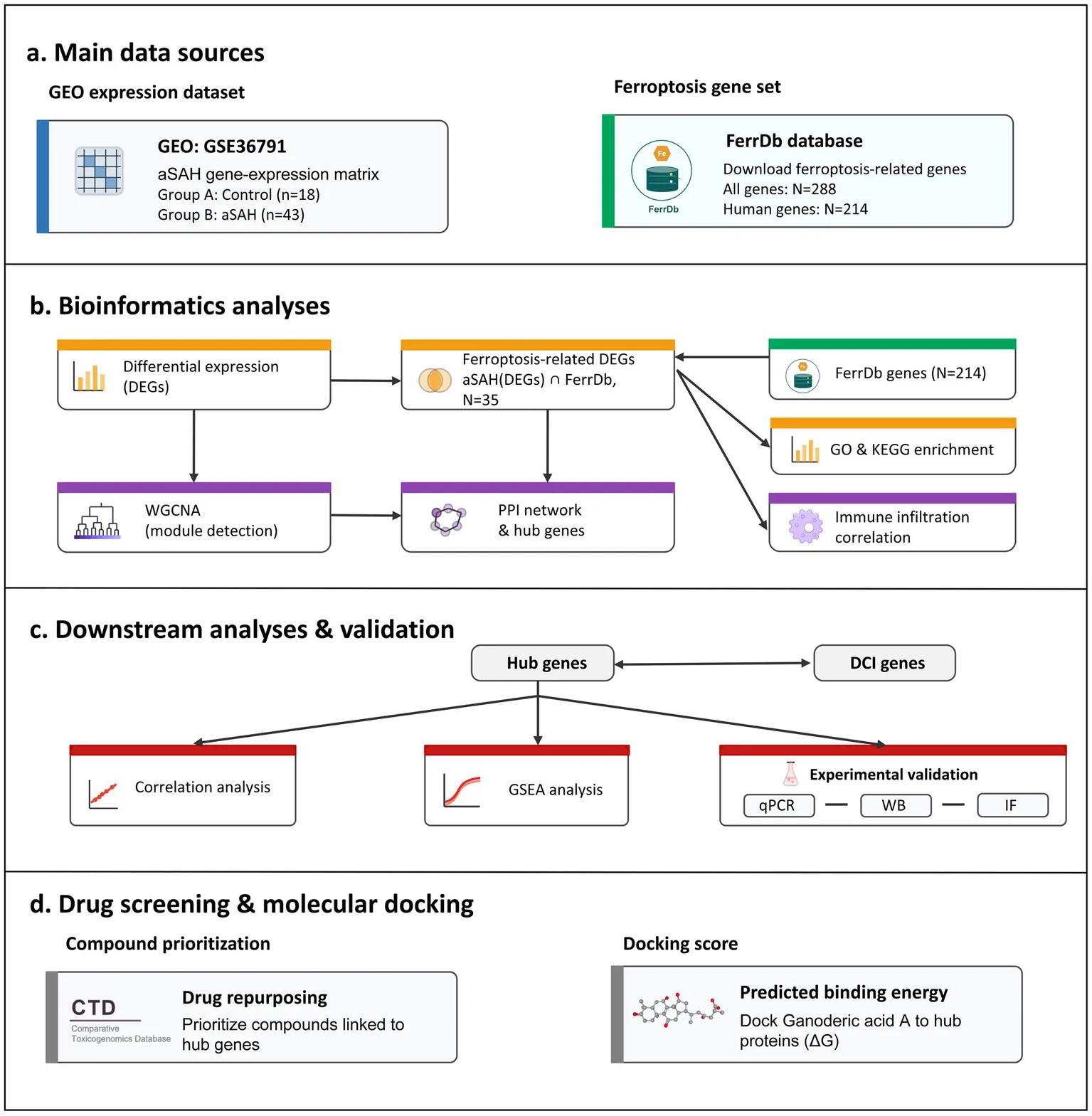
Overview of the study workflow. **(A)** Main data sources. **(B)** Bioinformatics analyses. **(C)** Downstream analyses and validation. **(D)** Compound prioritization and molecular docking.

### Single-cell RNA-seq data processing

The OMIX006611 single-cell RNA-seq dataset was analyzed using the Scanpy package in Python (15). The raw 10x Genomics count matrix was first imported. For quality control, cells were retained if they had ≥ 200 detected genes, ≤ 15% mitochondrial gene content, and ≤ 3% hemoglobin-related gene content. Doublets were then detected using Scrublet, and cells with a doublet score > 0.06 were removed (16). After quality control, the raw count matrix was normalized to 10,000 counts per cell and then log1p-transformed (17). The top 3,000 highly variable genes were selected using the Seurat v3 method, and ribosomal protein genes beginning with Rpl or Rps were excluded. For dimensionality reduction, PCA was performed based on the scaled highly variable gene matrix, and the first 50 principal components were calculated. A nearest-neighbor graph was constructed based on the PCA results, and UMAP visualization was performed using the first 25 principal components, with min_dist = 0.25 and random_state = 0 (18). Cell clustering was performed using the Leiden algorithm. Multiple resolution parameters, including 0.25, 0.5, 0.75, and 1.0, were evaluated, and the clustering result at resolution = 0.5 was selected for subsequent cell-type annotation and candidate gene expression analysis.

### WGCNA analysis of GEO

We constructed a weighted gene co-expression network in WGCNA (R) (19). The 5,000 genes with the largest median absolute deviation were analysed. Using pickSoftThreshold, a soft-threshold β = 8 was chosen to approximate a scale-free topology. The resulting adjacency matrix was converted to a topological-overlap matrix (TOM), and genes were clustered into modules with minModuleSize = 25 by dynamic tree cutting. Each module was summarised by its eigengene and displayed with a distinct colour (Supplementary Table 1).

### Differentially expressed gene (DEG) analysis

DEGs analysis was conducted on the batch-corrected GSE36791 dataset using the “limma” software. The cut-off values selected to identify DEGs were |fold change (FC)| > 1.2 and *p* < 0.05.

### Functional enrichment analysis

Kyoto Encyclopedia of Genes (KEGG) and Gene Ontology were used to determine gene regulation and function. Biological Process (BP), Cellular Component (CC), Molecular Function (MF) analysis were included in the functional enrichment analysis. The functions are performed by the clusterProfiler package (20). We defined the critical criterion for statistical significance as a *p*-value < 0.05. Functional enrichment analysis results were visualized in R using the GOplot and ggplot packages.

### Immune cells infiltration analysis

The immune abundance of the samples was calculated using the CIBERSORT algorithm in the R software (21). The LM22 annotation file contains expression substrates for 22 immune cells, with their abundance calculated using the CIBERSORT R function. Additionally, one can use the ssGSEA algorithm as an alternative method to analyze immune infiltration (22). The GSVA R package calculates 28 immune cell counts within a single sample, with a critical criterion for statistical significance set at a *p*-value < 0.05. We analyzed outcomes that were determined to be statistically significant.

### GSEA analysis of the top 4 hub genes

Single-gene GSEA analysis in aSAH or non-SAH was performed using the “clusterProfiler” package after identifying the hub genes. Patients in the aSAH or control group were divided into high and low expression groups based on the median expression of the hub gene. GSEA was utilized to gauge the enrichment score of gene sets and detect distinct functional phenotypes. GSEA was also employed to compare the biological pathways between the two groups. Significant gene sets had nominal *p* values < 0.05, false positive rate (FDR) q values < 0.05, and normalized enrichment scores (NES) > 1.

### Clinical information

With the approval of the institutional ethics review board, we retrospectively included 20 individuals (Supplementary material S1), comprising 10 patients diagnosed with aSAH by digital subtraction angiography and 10 non-SAH controls. The aSAH group included 5 males and 5 females, with a mean age of 60.2 ± 7.7 years. The non-SAH control group included 4 males and 6 females, with a mean age of 63.6 ± 8.9 years. De-identified residual peripheral-blood samples obtained from routine clinical laboratory testing were used. Samples from patients with aSAH were obtained within 24 h after aneurysmal rupture. Clinical data were collected between July and October 2023 at the Department of Neurosurgery, Tianjin Medical University General Hospital, Tianjin, China.

### Quantitative real-time PCR (qPCR)

Total RNA was extracted using TRIzol reagent (Invitrogen, Carlsbad, CA) according to the manufacturer’s instructions from blood samples of patients in both the aSAH and control groups. The relative expression levels of MAPK3, STAT3, G6PDH, and PGD in comparison to GAPDH were calculated by the 2^ΔΔCt^ method. Primers for PCR were designed through a primer designing tool (http://www.ncbi.nlm.nih.gov/tools/primer-blast/) and can be found in Supplementary material S3.

### Statistical analyses

Statistical analyses were performed using R software, SPSS version 23.0 (IBM, USA), and GraphPad Prism version 8.5. Student’s t-test, the Mann–Whitney U test, the Wald test, and Spearman’s rank correlation analysis were used as appropriate. Where applicable, *p* values were adjusted using the Benjamini–Hochberg method. Data are presented as mean ± SEM, and *p* < 0.05 was considered statistically significant.

### Reagents

β-actin (RRID: AB_2863784), STAT3 (RRID: AB_2758462), p-STAT3 (RRID: AB_2863810), G6PD (RRID: AB_3675843), MAPK3/ERK1 (RRID: AB_2862668) and PGD (RRID: AB_3675844) were purchased from Abclonal (Wuhan, CHINA).

### Animals and SAH model

All procedures were approved by the Ethics Committee of Tianjin Medical University General Hospital (Approval No. IRB2025-DW-72) and followed ARRIVE recommendations. Male C57BL/6 J mice (8 weeks, 20–22 g; Beijing, China) were housed in SPF rooms on a 12-h light/dark schedule with controlled temperature and humidity; food and water were available ad libitum. For experimental validation, mice were assigned to the sham and SAH groups, with 6 mice in each group.

Subarachnoid hemorrhage (SAH) was produced by cisterna magna autologous blood injection. Sixty microliters of freshly collected arterial blood was infused using a microsyringe at 30 μL·min^−1^; the needle remained *in situ* for 3 min to limit reflux. Animals recovered on a heating pad with the head tilted ~30° downward to facilitate subarachnoid distribution of blood. Sham mice underwent identical exposure without blood infusion (23). Brain tissues were collected 1 day after SAH or sham operation for Western blotting, immunofluorescence staining, MDA measurement, and GPX4 activity assays.

### Ferroptosis-related biochemical assays

MDA content and GPX4 activity were measured to evaluate ferroptosis-related biochemical changes in mouse brain tissue. Brain tissues were homogenized on ice and centrifuged at 10,000 × g for 10 min at 4 °C. The supernatants were collected for subsequent assays, and protein concentrations were determined for normalization. MDA content was measured using a lipid peroxidation MDA assay kit (S0131, Beyotime, Shanghai, China). The assay is based on the reaction between MDA and thiobarbituric acid, which generates a colored MDA-TBA adduct. Absorbance was measured at 532 nm, and MDA levels were calculated according to the standard curve and normalized to protein concentration. GPX4 activity was measured using a glutathione peroxidase 4 activity assay kit (BC6260, Solarbio, Beijing, China). The assay was performed according to the manufacturer’s instructions by monitoring the decrease in NADPH absorbance at 340 nm. GPX4 activity was calculated according to the manufacturer’s formula and normalized to protein concentration. MDA levels were expressed as nmol/mg protein, and GPX4 activity was expressed as U/mg protein.

### Western blotting (WB)

Proteins were extracted from brain tissue in ice-cold RIPA buffer (Beyotime, P10013B) supplemented with protease and phosphatase inhibitors (Epizyme, GRF101/GRF102). Concentrations were measured by BCA (Beyotime, P0012S). Equal protein amounts (20 μg) were separated by SDS–PAGE and transferred to PVDF membranes (Millipore, 1,620,177/1620264). Membranes were blocked in 5% skim milk in TBST (Yase, PS103) for 2 h at room temperature, incubated overnight at 4 °C with primary antibodies (β-actin 1:10,000; others 1:1,000), washed, and then probed with HRP-conjugated secondary antibodies (ZSGB-BIO, 1:5,000, 1 h, RT). Signals were developed by ECL and imaged on a ChemiDoc Touch (Bio-Rad). Band intensities were quantified using ImageJ v1.53 after background subtraction. p-STAT3 was normalized to total STAT3, while STAT3, PGD, ERK1/MAPK3, and G6PD were normalized to β-actin and expressed relative to the sham group.

### Immunofluorescence imaging

Immunostaining was performed using previously described methods. Briefly, mice were deeply anesthetized and perfused transcardially with phosphate-buffered saline (PBS) followed by 4% paraformaldehyde (PFA). Brains were harvested, post-fixed in 4% PFA at 4 °C overnight, and dehydrated through a graded sucrose series. Frozen coronal sections were rinsed in PBS and blocked/permeabilized for 1 h at room temperature in PBS containing 0.3% Triton X-100 and 5% bovine serum albumin (BSA). Sections were then incubated overnight at 4 °C with the following primary antibodies: anti-PGD (1:100, Cat. A0563, Abclonal, Wuhan, China), anti-STAT3 (1:100, Cat. A11216, Abclonal), anti-MAPK3/ERK1 (1:100, Cat. A19561, Abclonal), and anti-G6PD (1:100, Cat. A27296, Abclonal). After three PBS washes, sections were exposed to the appropriate Alexa Fluor-conjugated secondary antibodies for 1 h at room temperature. Nuclei were counterstained with 4′,6-diamidino-2-phenylindole (DAPI; Sigma-Aldrich, St. Louis, MO, USA). Fluorescent signals were captured using an Olympus BX-61 fluorescence microscope (Olympus, Tokyo, Japan) and quantified with ImageJ (NIH) to assess fluorescence intensity and distribution.

### Drug screening and molecular docking

Four ferroptosis-related DEGs were selected as therapeutic targets. Corresponding small-molecule interactors were retrieved from the Comparative Toxicogenomics Database (CTD, https://ctdbase.org/), and their 3-D coordinates were downloaded from the Protein Data Bank(PDB, https://rcsb.org/) (or standardised conformers from PubChem if unavailable). High-resolution protein structures were obtained from UniProt (https://uniprot.org/), with AlphaFold models used when no experimental structure existed. AutoDock Vina was employed for molecular docking, using a grid box centred on each protein’s canonical binding pocket. For every ligand–protein pair, the lowest-energy pose (Vina score, kcal mol^−1^) was retained. Docked complexes were visualised in PyMOL to examine binding orientation, hydrogen-bond networks, hydrophobic contacts, and key interacting residues for mechanistic insight.

## Results

### Data normalisation and DEGs analysis

For this study, an expression analysis (GSE36791) was performed from the GEO database. The GSE36791 dataset was first processed using the limma package normalise BetweenArrays processing in R software (Figure 2A), and the PCA results (Figure 2B) showed that there was good biological replication consistency across biological samples, and the 18 control and 43 ruptured aneurysm (aSAH) groups within the GSE36791 dataset were peripheral blood samples were analysed for differentially expressed genes. The R package limma was run for differentially expressed genes. Post-screening analysis with a threshold of |FC| > 1.2 and a *p*-value < 0.05 found 1,097 up-regulated genes and 1,548 down-regulated genes in blood samples from SAH blood samples. Figure 2C displays a volcano map while Figure 3A shows a heat map with all DEGs.

**Figure 2:**
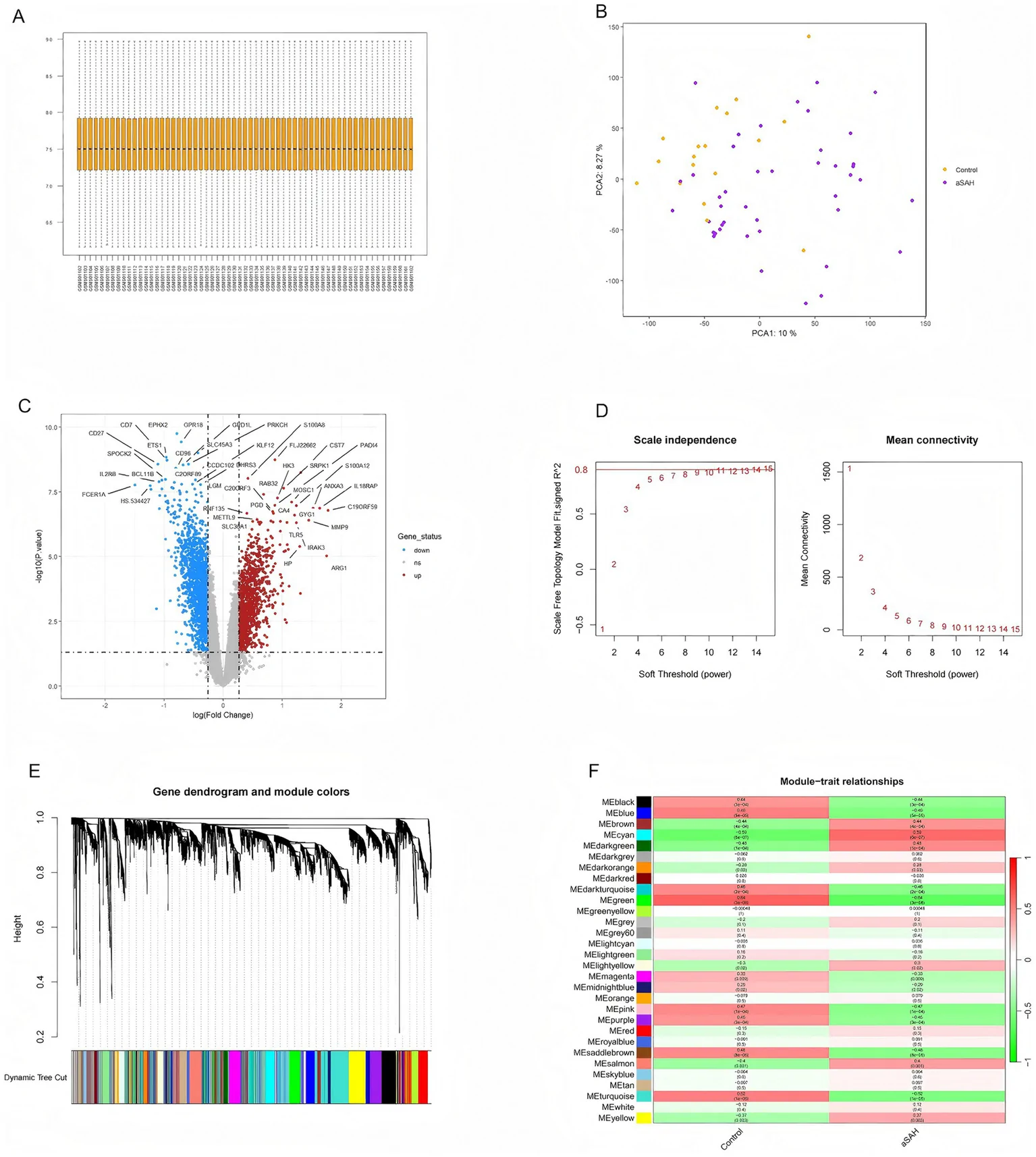
Data filtering and identification of DEGs in the SAH blood samples. **(A)** Box plot, GSE36791 normalized data. **(B)** PCA analysis of the GSE36791 aSAH group and the control group. **(C)** The volcano plot. Red dots represent up-regulated genes, grey dots represent not significant genes, and blue dots represent down-regulated genes. **(D)** Determination of the soft-thresholding power for WGCNA. The red line indicates the approximate scale-free topology fit index threshold of 0.8, and *β* = 8 was selected for network construction. **(E)** WGCNA analysis cluster dendrogram of co-expression genes in GSE36791. **(F)** Module–trait relationship heatmap showing correlations between module eigengenes and sample status (control or aSAH), with corresponding p values. PCA, principal component analysis; DEGs, differentially expressed genes; WGCNA, weighted gene co-expression network analysis.

**Figure 3:**
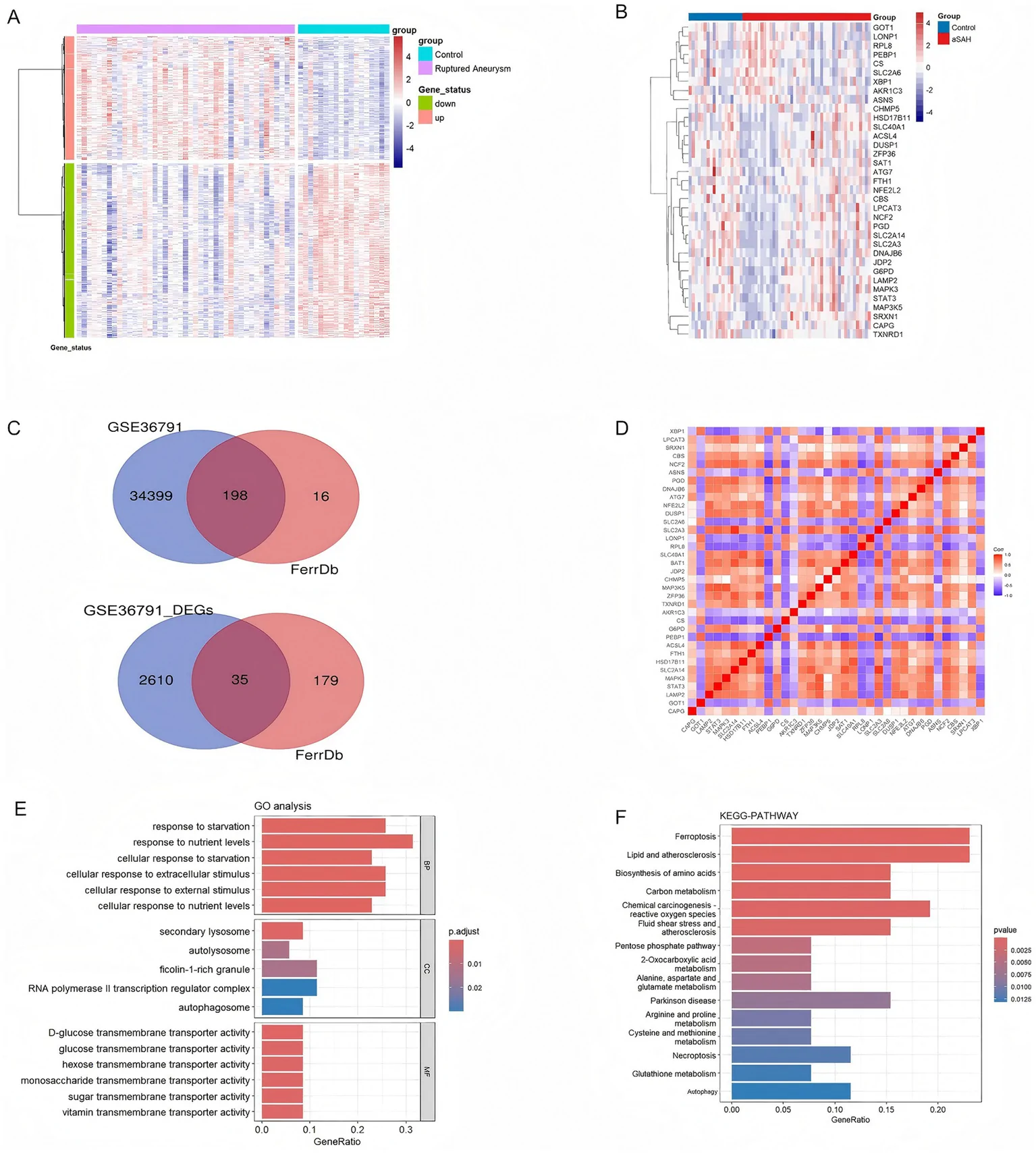
Identification and functional enrichment of ferroptosis-related differentially expressed genes in the GSE36791 peripheral-blood dataset. **(A)** Heatmap of all differentially expressed genes in control and aSAH samples. **(B)** Heatmap of the 35 ferroptosis-related differentially expressed genes. **(C)** Venn diagrams showing the overlap between all genes detected in GSE36791 and human ferroptosis-related genes obtained from FerrDb, and the overlap between GSE36791 differentially expressed genes and FerrDb genes, identifying 35 ferroptosis-related differentially expressed genes. **(D)** Spearman correlation heatmap of the 35 ferroptosis-related differentially expressed genes. **(E)** Gene Ontology enrichment analysis of these genes. **(F)** Kyoto Encyclopedia of Genes and Genomes pathway enrichment analysis of these genes. DEGs, differentially expressed genes; Ferr-DEGs, ferroptosis-related differentially expressed genes; GO, Gene Ontology; KEGG, Kyoto Encyclopedia of Genes and Genomes.

### WGCNA and key module identification

We performed WGCNA to analyse the GSE36791 dataset to obtain key modules and genes, taking the top 5,000 genes in terms of median absolute deviation (MAD) applied to the identified highly relevant gene modules(Figures 2D,E). The first-class soft threshold for this model was set at 8 using the selection soft threshold function. The topological scale-free fit index reached 0.8. After calculating the weighting coefficients, we obtained a disTOM consisting of 5,000 genes, delineating 30 modules, each uniquely represented by a color. The heat map indicates the correlation between the module eigenvalues and aSAH, with each column exhibiting the correlation coefficient and the related *p*-value. Red indicates a positive correlation, and green shows a negative correlation. The deeper the color, the stronger the correlation coefficient. Figure 2F displays the clustering and correlation of the featured genes in each module. It is evident that the cyan and dark green modules exhibit the highest positive correlation with aSAH, with correlation coefficients of 0.59 and 0.48, respectively. Conversely, the green and turquoise modules show the highest negative correlation with aSAH, with correlation coefficients of −0.64 and −0.52, respectively. This suggests that the genes within these modules have high modularity properties and correlation with aSAH.

### Ferr-DEGs validation and enrichment analysis

The Venn diagram indicates that 198 genes in GSE36791 overlap with FerrDb, of which 35 are ferroptosis-related differentially expressed genes (Figure 3C). Peripheral-blood transcriptomic profiles for ferroptosis-related genes (FRGs) in aSAH patients are presented as a heat-map (Figure 3B and Supplementary material S2). Relative to healthy controls, canonical pro-ferroptotic drivers, such as ACSL4, FTH1, STAT3, and MAPK3, are broadly up-regulated, whereas several antioxidant or inhibitory FRGs exhibit relative down-regulation. Spearman correlation analysis showed close associations among the Ferr-DEGs, as shown in Figure 3D. GO enrichment analysis indicated that these genes were mainly involved in stress responses, metabolic homeostasis, autophagy-related components, oxidoreductase activity, and nutrient metabolism (Figure 3E). KEGG analysis further showed enrichment in ferroptosis, lipid and atherosclerosis, and amino acid biosynthesis pathways (Figure 3F). These results suggest that Ferr-DEGs are associated with oxidative stress and metabolic remodeling in peripheral blood from patients with aSAH. These enriched pathways, however, may reflect general oxidative and inflammatory responses rather than being exclusively specific to aSAH.

### Identifying disparities in immune infiltration between aSAH and control group

To characterize immune infiltration in peripheral blood from patients with aSAH, we analyzed the data by using the CIBERSORT and ssGSEA algorithms. CIBERSORT results indicated higher levels of neutrophils, activated mast cells, and activated dendritic cells in aSAH blood samples compared to blood samples (Figure 4A). The sample-level distribution of CIBERSORT-estimated immune-cell fractions is shown in Figure 4B. The ssGSEA results revealed a similar trend to that of CIBERSORT analysis (Figure 4C). Furthermore, Spearman correlation analysis showed associations between Ferr-DEGs and ssGSEA-derived immune-cell scores (Figure 4D). Various immune cells showed differential infiltration in the two groups of blood samples. However, these immune changes may also represent general systemic responses to acute injury or stress, thus requiring additional studies to confirm their specificity for aSAH.

**Figure 4:**
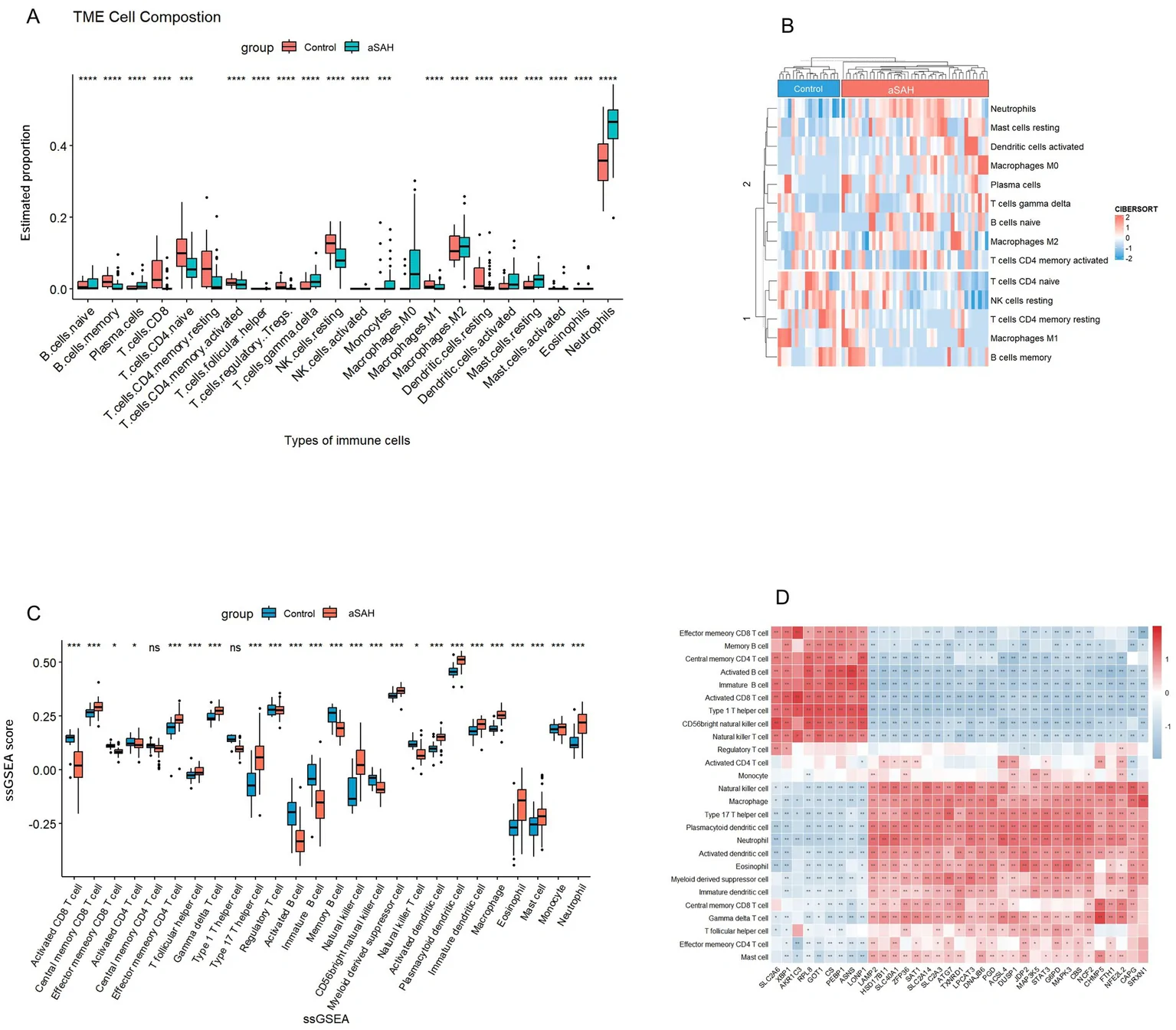
Peripheral immune-cell composition analysis in aSAH and control blood samples. **(A)** Boxplot of CIBERSORT-estimated proportions of 22 immune-cell type. **(B)** Heatmap of CIBERSORT-estimated immune-cell proportions across samples. **(C)** Boxplot of ssGSEA-derived immune-cell signature scores between aSAH and control samples. **(D)** Spearman correlation heatmap between Ferr-DEGs and ssGSEA-derived immune-cell scores.

### Ferr-DEGs hub gene identification

Ferr-DEG were uploaded to the STRING database to retrieve the PPI networks (Figures 5A,B). The PPI networks were constructed through Cytoscape. Using the cytoHubba plugin, ten hub genes were identified, which included PGD, FTH1, SLC40A1, STAT3, TXNRD1, ACSL4, MAPK3, G6PD, NFE2L2, SAT1. Next, the PPI network was analysed using the MCODE plugin,which identified three densely connected clusters with scores of 4.286, 4.000, and 3.000, respectively. MCODE 1 included FTH1, PGD, SRXN1, SLC40A1, TXNRD1, LPCAT3, ACSL4, SAT1. MCODE 2 included XBP1, ATG7, MAPK3, STAT3. MCODE 3 Includes G6PD, CS, GOT1 (Figures 5C–E). Ultimately, PGD, FTH1, SLC40A1, STAT3, TXNRD1, ACSL4, MAPK3, G6PD, and SAT1 play important roles in both CytoHubba and MCODE analyses. Finally, after intersecting the Cytoscape-derived hub genes with genes from the four WGCNA modules, including two positively correlated and two negatively correlated modules, four hub genes were identified: STAT3, G6PD, MAPK3, and PGD (Figure 5F). To determine whether the four hub genes were specific to aneurysmal subarachnoid hemorrhage (aSAH) or reflected a broader acute cerebrovascular injury response, we further analyzed peripheral-blood datasets from patients with ischemic stroke and traumatic brain injury (Supplementary Figure 1). Compared with controls, G6PD, MAPK3, PGD, and STAT3 were all significantly upregulated in peripheral blood cells from patients with ischemic stroke and traumatic brain injury. These results suggest that the four hub genes are not exclusively specific to aSAH, but may represent a shared peripheral stress, inflammatory, and metabolic response to acute cerebrovascular injury (13, 14, 24).

**Figure 5:**
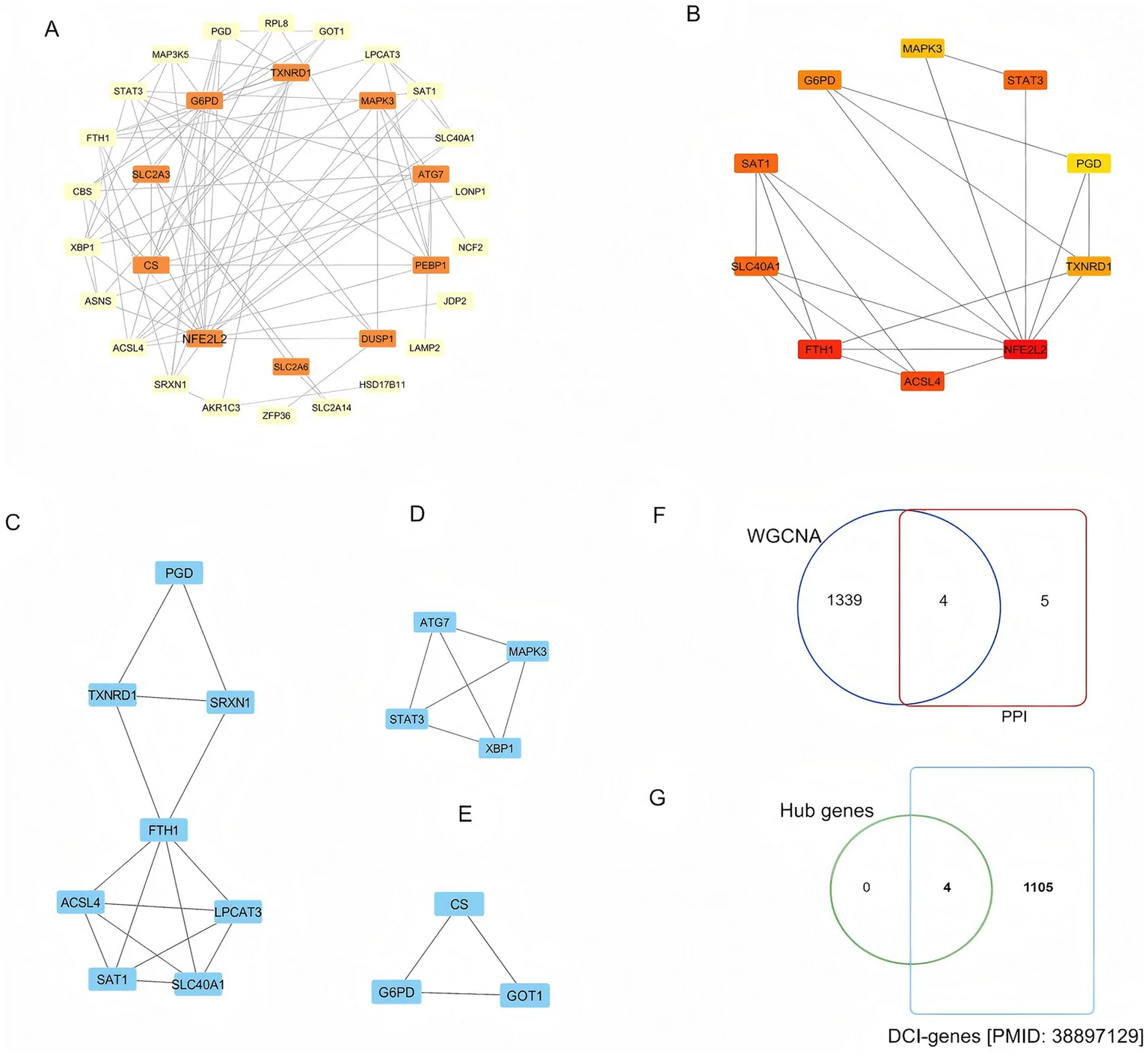
Network-based identification of ferroptosis-associated candidate hub genes in aSAH. **(A)** PPI network constructed from ferroptosis-related DEGs. **(B)** Top-ranked PPI hub genes identified by cytoHubba. **(C–E)** MCODE clusters identified from the PPI network, with scores of 4.286, 4.000, and 3.000, respectively. **(F)** Intersection between WGCNA module genes and PPI-derived hub genes. **(G)** Overlap between the four selected hub genes and DCI-associated genes. PPI, protein–protein interaction; DCI, delayed cerebral ischemia.

### Expression of key hub genes in samples and GSEA analysis

We analysed the first 4 hub genes using GSEA with a *p*-value < 0.05. After analyzing the pathways in which each hub gene’s expression profile is located using the KEGG pathway database, we found that the enriched pathways primarily centered on Ribosome (Supplementary Figure 2).

### Validation of ferroptosis-related hub genes in aSAH

The expression of the four hub genes was first assessed in the GSE36791 human peripheral-blood dataset. STAT3, MAPK3, G6PD, and PGD showed higher expression in aSAH samples than in controls (Figure 6A). Importantly, all four final candidate genes remained significantly upregulated after Benjamini–Hochberg FDR correction in the GSE36791 dataset: PGD, adjusted *p* = 9.96 × 10^−5^; G6PD, adjusted *p* = 0.00957; MAPK3, adjusted *p* = 0.01284; and STAT3, adjusted *p* = 0.01831. To further evaluate whether these peripheral-blood candidates were supported in brain tissue, we analyzed the GSE79416 mouse cortex dataset. The corresponding mouse orthologs Mapk3, Stat3, G6pdx, and Pgd were also significantly upregulated in SAH cortex compared with control cortex (Figure 6B). qPCR analysis in the clinical peripheral-blood cohort further confirmed increased MAPK3, STAT3, PGD, and G6PD expression in patients with aSAH compared with controls (Figure 6C). Public mouse SAH single-cell RNA-seq analysis identified major brain and immune cell populations across different time points (Figure 6D). Single-cell expression analysis showed that Mapk3, Stat3, G6pdx, and Pgd were detectable across annotated brain cell types, with relatively evident expression in immune/glial cell populations (Figure 6E). These results provide cross-dataset support for the four ferroptosis-related hub genes at the peripheral-blood, mouse cortex, and single-cell levels.

**Figure 6:**
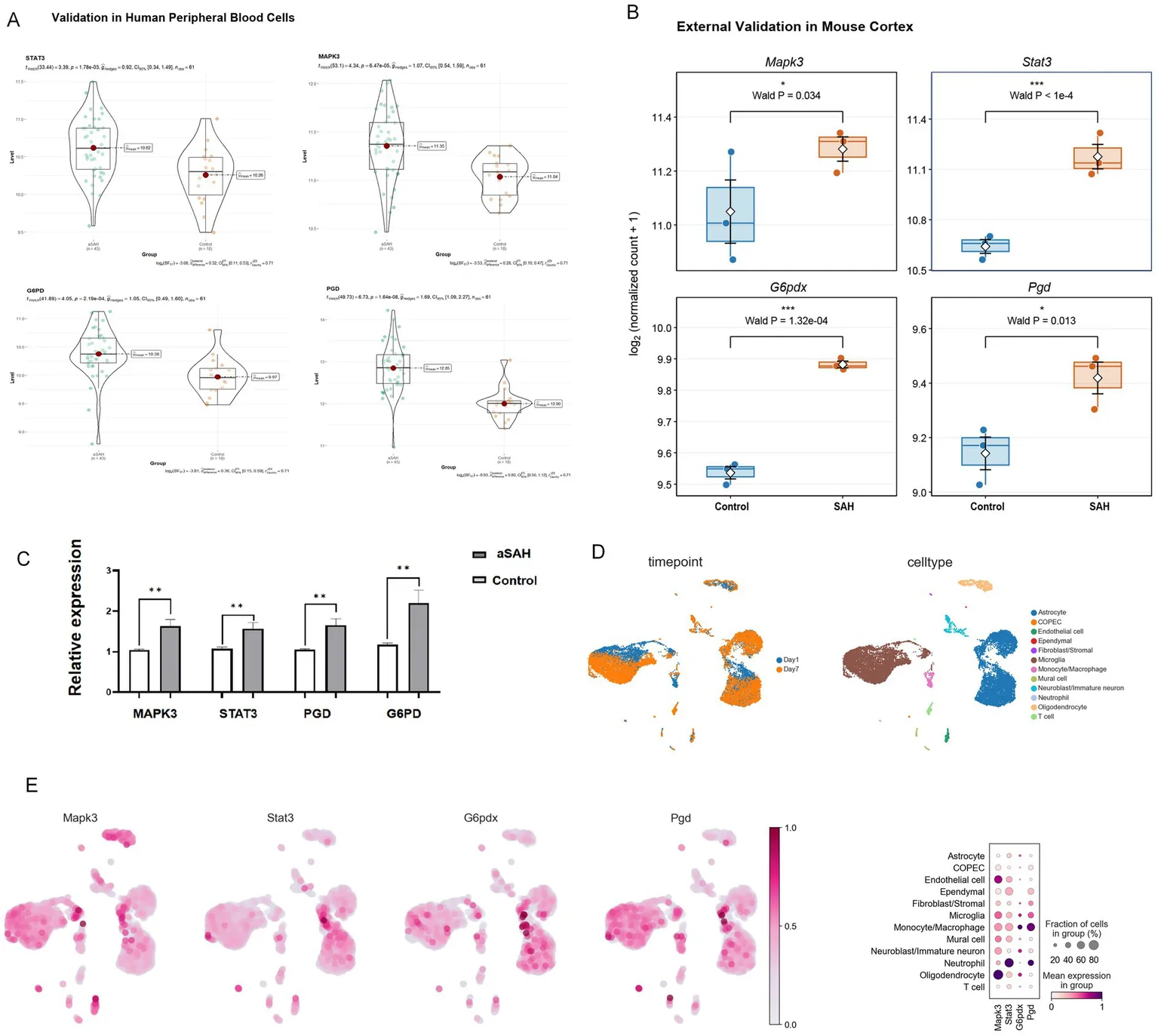
Cross-dataset and experimental validation of ferroptosis-associated candidate hub genes. **(A)** Violin plots comparing STAT3, MAPK3, G6PD, and PGD expression between control and aSAH samples in the GSE36791 human peripheral-blood dataset. **(B)** Boxplots comparing Mapk3, Stat3, G6pdx, and Pgd expression between control and SAH mouse cortex samples in the GSE79416 dataset; Wald p values are shown. **(C)** Quantitative real-time PCR validation of MAPK3, STAT3, PGD, and G6PD mRNA expression in the clinical peripheral-blood cohort. **(D)** Uniform manifold approximation and projection plots of the OMIX006611 mouse SAH single-cell RNA-sequencing dataset, colored by time point and annotated cell type. **(E)** UMAP feature plots and a dot plot showing Mapk3, Stat3, G6pdx, and Pgd expression across annotated cell types. qPCR, quantitative real-time PCR; UMAP, uniform manifold approximation and projection.

Validation of hub-gene protein expression and ferroptosis-associated biochemical changes in SAH mouse brain.

To determine whether the transcriptomic hub genes were supported at the protein level in brain tissue, we examined MAPK3/ERK1, STAT3, PGD, and G6PD expression in a mouse model of subarachnoid hemorrhage (SAH). Western blotting showed that total STAT3, PGD, G6PD, and MAPK3/ERK1 protein levels were significantly increased in cortical lysates from SAH mice compared with sham-operated controls (Figures 7A,B). In addition, phosphorylated STAT3 was elevated, as indicated by an increased p-STAT3/STAT3 ratio, suggesting activation of STAT3 signaling after SAH (Figures 7A,B). To further assess whether these protein changes were accompanied by ferroptosis-associated biochemical alterations, we measured lipid peroxidation and GPX4 activity in mouse brain tissue. SAH mice showed significantly increased MDA levels and reduced GPX4 activity compared with sham mice, indicating enhanced lipid peroxidation and impaired GPX4-dependent antioxidant defense after SAH (Figure 7C). Immunofluorescence staining further confirmed increased STAT3-, G6PD-, PGD-, and MAPK3-positive signals in SAH brain tissue compared with sham controls (Figures 7D,E). Together, these results provide protein-level support for the four hub genes and indicate that experimental SAH is accompanied by ferroptosis-associated biochemical changes in brain tissue.

**Figure 7:**
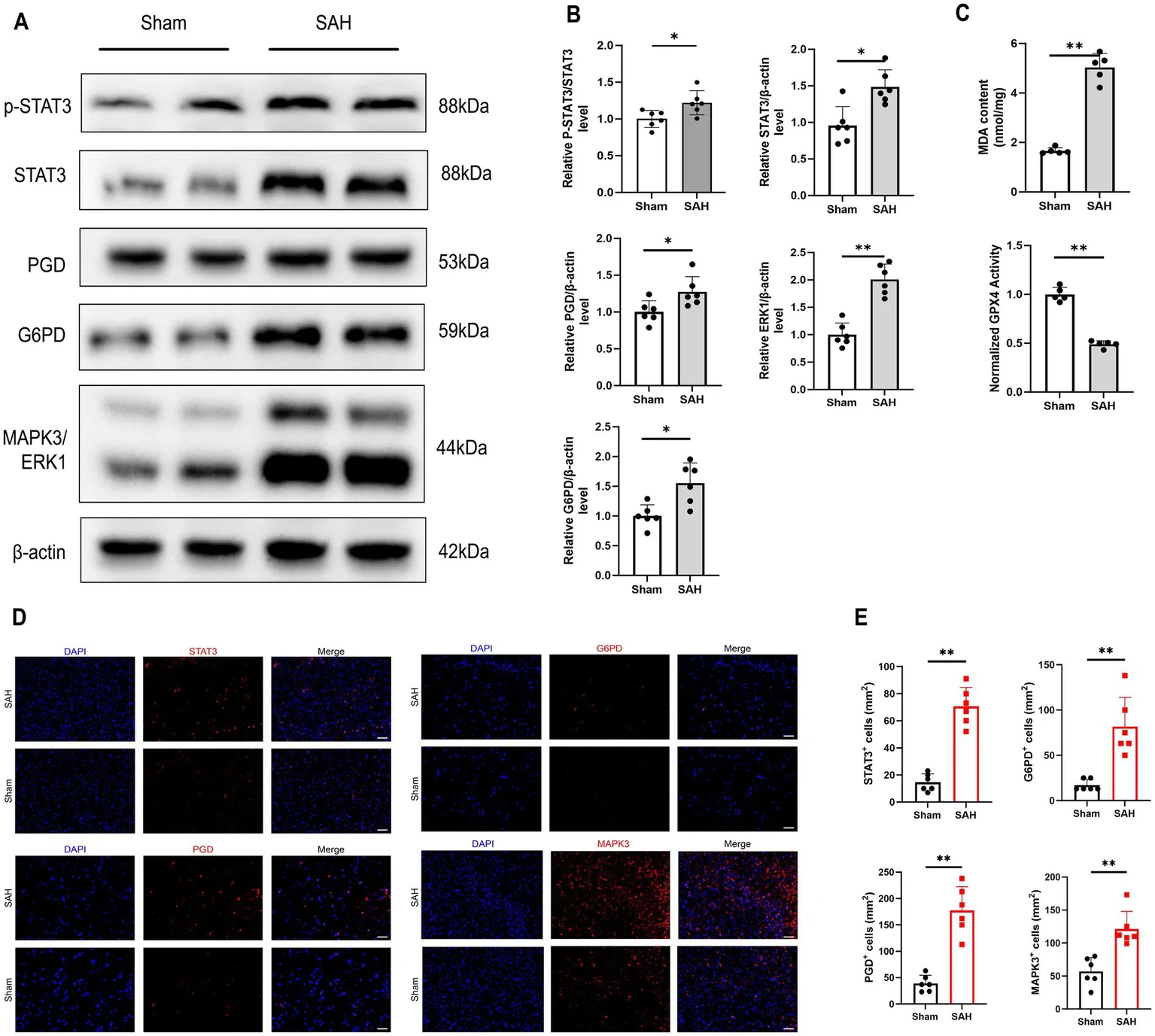
Protein-level validation of hub genes and ferroptosis-associated biochemical changes in SAH mouse brain. **(A)** Representative Western blot bands of p-STAT3, total STAT3, PGD, G6PD, MAPK3/ERK1, and β-actin in brain tissue from sham and SAH mice. **(B)** Quantitative analysis of Western blot bands. p-STAT3 was normalized to total STAT3, whereas total STAT3, PGD, G6PD, and MAPK3/ERK1 were normalized to β-actin. **(C)** MDA content and GPX4 activity in mouse brain tissue. SAH mice showed increased MDA levels and reduced GPX4 activity compared with sham mice. **(D)** Representative immunofluorescence images showing STAT3, G6PD, PGD, and MAPK3 expression in brain sections from sham and SAH mice. Nuclei were counterstained with DAPI. **(E)** Quantification of STAT3-, G6PD-, PGD-, and MAPK3-positive cells in brain tissue. Data are shown as mean ± SEM. Each dot represents one biological replicate. Statistical significance was assessed using a two-tailed Mann–Whitney U test. *p < 0.05, **p < 0.01 versus sham.

### Small-molecule prioritization and molecular docking analysis

A Venn diagram was employed to visualise the overlap between four hub genes, PGD, MAPK3, G6PD and STAT3, and their corresponding small-molecule interaction networks. Ganoderic acid A was prioritized as a potential multi-target therapeutic candidate predicted to interact with PGD, MAPK3, G6PD, and STAT3 (Figure 8A). Detailed docking assessments of ganoderic acid A with each protein (Figures 8B–E) yielded the following binding free energies: PGD, −9.0 kcal mol^−1^; MAPK3, −8.8 kcal mol^−1^; G6PD, −7.6 kcal mol^−1^; and STAT3, −7.5 kcal mol^−1^. Collectively, these data indicate that ganoderic acid A exhibits strong binding affinity toward all four Ferr-DEG-encoded proteins, with particularly favourable interactions observed for PGD and MAPK3 (binding energy < −8.8 kcal mol^−1^). These findings suggest that ganoderic acid A may exert therapeutic effects by simultaneously modulating multiple ferroptosis-associated pathways, providing a molecular rationale for its further investigation in disease-relevant models.

**Figure 8:**
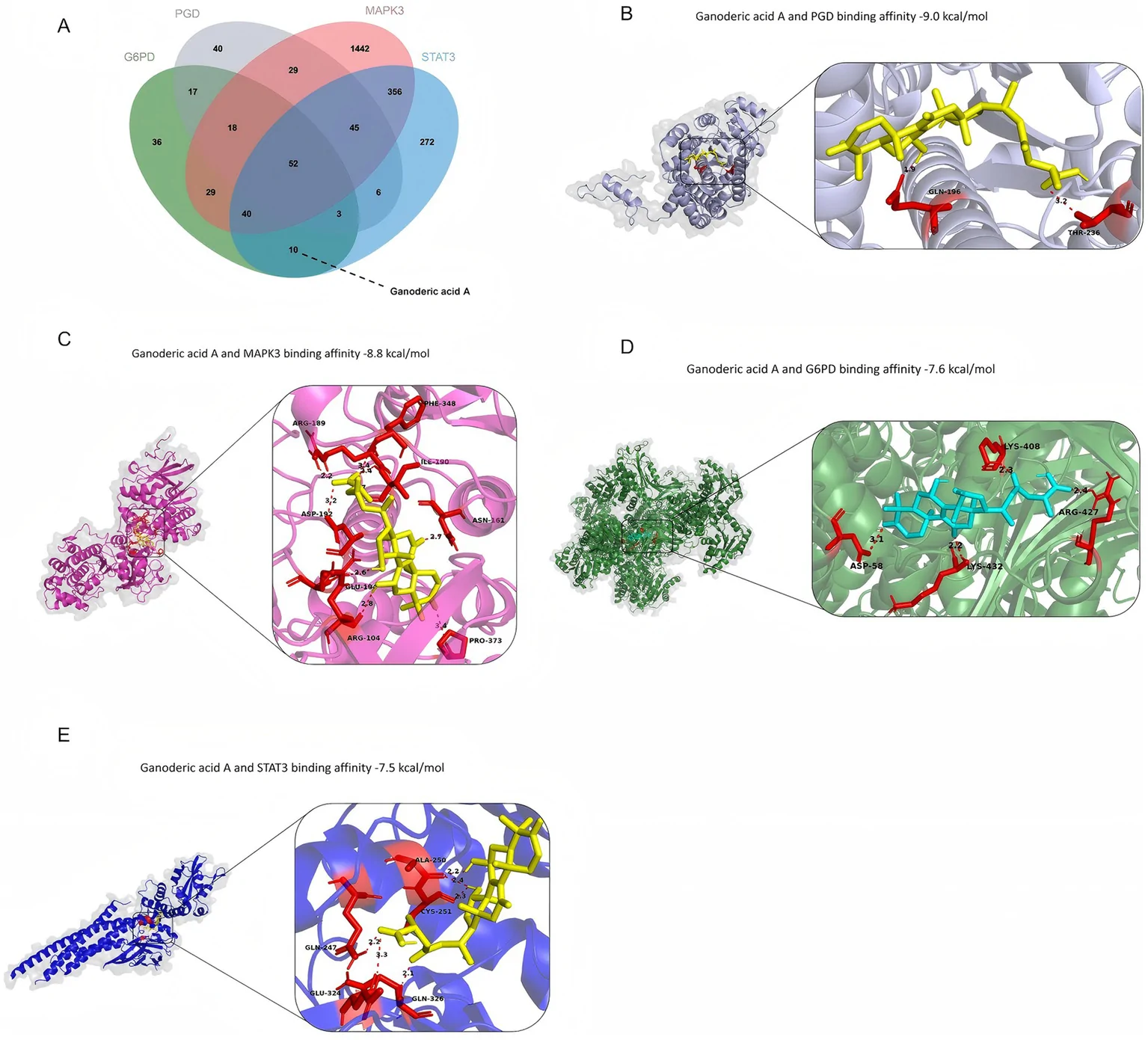
CTD-based compound prioritization and molecular docking of ganoderic acid A. **(A)** Venn diagram of CTD-associated compounds for PGD, MAPK3, G6PD, and STAT3, highlighting ganoderic acid A. **(B–E)** Predicted docking poses of ganoderic acid A with PGD, MAPK3, G6PD, and STAT3, respectively. AutoDock Vina scores were −9.0, −8.8, −7.6, and −7.5 kcal/mol, respectively. CTD, Comparative Toxicogenomics Database.

## Discussion

Many experimental studies on EBI-aSAH have shown that neuronal cell death is the major cause of neurological dysfunction after aSAH. As ferroptosis is closely related to early brain injury (EBI) after aSAH, timely detection and inhibition of ferroptosis onset are crucial for aSAH prognosis (25). Ferroptosis is a type of iron-dependent, regulatory necrosis that is marked by the retention of intracellular iron, reduction of GSH, and the buildup of lipid ROS (26). As levels of reactive oxygen species (ROS) increase, they cause oxidative stress injury and ultimately lead to cell death. This is a crucial pathological mechanism in the development of EBI after aSAH (27, 28). Due to the rapid accumulation free iron in the subarachnoid hemorrhage region after rupture of intracranial aneurysms, many studies have recognized the critical role of ferroptosis in aSAH. The utilization of iron chelators, which can effectively inhibit cytosolic iron accumulation and oxidative stress, may be neuroprotective and ameliorate the adverse outcome of aSAH (29–31).

We identified 2,645 DEGs from the GSE36791 dataset, including 1,097 up-regulated and 1,548 down-regulated genes. Our results suggest that ferroptosis may play a role in the pathogenesis of aSAH; however, further research is needed to determine whether the identified hub genes are specific to aSAH or represent general stress-related genes. We obtained 35 overlapping genes after comparing DEG and FerrDb. GO and KEGG enrichment analyses revealed their strong association with oxidative stress and ferroptosis. Using WGCNA and PPI network analyses, four hub genes were identified as STAT3, G6PD, MAPK3 and PGD.

To further evaluate the specificity of these four hub genes, we analyzed peripheral-blood datasets from patients with ischemic stroke and traumatic brain injury. G6PD, MAPK3, PGD, and STAT3 were also upregulated in these acute neurological injury datasets, suggesting that these genes are not exclusively specific to aSAH, but may represent a shared peripheral stress, inflammatory, and metabolic response to acute neurological injury. In addition, because the discovery dataset was derived from peripheral blood, we further analyzed the GSE79416 mouse cortex dataset and public mouse SAH single-cell RNA-seq data. The corresponding mouse orthologs Mapk3, Stat3, G6pdx, and Pgd were upregulated in SAH cortex, and single-cell analysis showed that these genes were detectable across annotated brain cell types, especially in immune/glial cell populations. These results provide supportive cross-dataset evidence linking the peripheral-blood findings to brain tissue responses, although they do not prove that peripheral blood expression directly reflects brain expression.

In this study, we analyzed the diverse expression of the top four hub genes in blood samples utilizing qPCR. STAT3(Signal transducer and activator of transcription 3) is a transcription factor found in the cytoplasm that becomes activated upon exposure to inflammatory cytokines or growth factors. It subsequently translocates to the nucleus where it plays a role in regulating DNA transcription. STAT3 has become a popular research topic as it is involved in various biological processes such as differentiation, proliferation, anti-apoptosis, inflammation and angiogenesis. It has been shown that STAT3 expression can exacerbate inflammation-induced ferroptosis, leading to a poor prognosis of aSAH (32–34). Yamamoto et al. reported that rapamycin is available as a novel pharmacological strategy to activate STAT3 for the treatment of EBI after aSAH (35).

MAPK3 (mitogen-activated protein kinase 3) is a member of the MAPKinase family of proteins that phosphorylate target proteins in a variety of cellular functions such as proliferation, inflammation, and cellular metabolism (36). MAPK3 has been shown to be associated with activation of the ERK pathway, which affects neural differentiation and proliferation. However, the specific mechanism for activating aSAH ferroptosis remains unknown and requires further investigation (37).

G6PD (glucose-6-phosphate dehydrogenase) is a crucial enzyme in the pentose phosphate pathway (PPP), and some research implies that G6PD deficiency decelerates iron transformation in HCC cells, which subsequently restrains tumor progression *in vivo* (38). Luo and his colleagues reported that upregulation of G6PD was associated with neuronal protection after aSAH, and by observing the short- and long-term neurobehavioural outcomes in EBI-aSAH rats, it was suggested that the elevation of G6PD was synchronous with the reduction of neuronal damage, which may be related to antioxidant defence and DNA repair (5).

PGD (phosphogluconate dehydrogenase) is also involved in energy metabolism and plays a role in the PPP. Dubreuil discovered that PGD knockdown disrupting the PPP suppressed ROS buildup (39, 40). Further research is necessary to investigate the correlation between aSAH and ferroptosis in PGD.

All four hub genes overlap with the delayed cerebral ischemia (DCI)–associated gene set and are poised to act synergistically through several converging mechanisms, iron-homeostasis disruption that increases labile iron, ACSL4-driven amplification of lipid peroxidation, GPX4/SLC7A11-mediated antioxidant collapse, and STAT3/MAPK3-dependent regulation of inflammatory and vasoreactive pathways (24, 41). Together these alterations promote microvascular vasospasm, microthrombosis, and blood–brain barrier breakdown during the vulnerable 3- to 14-day window after SAH, thereby driving the onset and progression of DCI (42). Notably, STAT3, MAPK3, G6PD, and PGD are also involved in multiple stress and inflammatory pathways, indicating that these genes may not be exclusively specific to ferroptosis in aSAH, and their precise roles warrant further investigation.

The pathological progression of aneurysmal subarachnoid hemorrhage (aSAH) significantly depends on immune response-related biological processes (4, 43). The inflammatory response triggered by aSAH follows a clearly defined timeline: Acute phase (≤72 h): marked by robust innate immune activation, rapid microglial activation, and substantial recruitment of neutrophils and monocytes/macrophages. Cytokines, including IL-6, TNF-α, and IL-10, surge quickly, resulting in peak inflammatory activity typically associated with poorer clinical outcomes. Subacute phase (days 3 to 14): inflammation undergoes progressive changes, coinciding with heightened risks of vasospasm and delayed cerebral ischemia (DCI) (44). Recovery phase (≥14 days): inflammation gradually diminishes, though chronic complications such as hydrocephalus and persistent immune dysfunction may remain (42).

At the cellular level, neutrophils dominate the acute phase, mobilizing massively within 24 h post-hemorrhage (45). While helpful in clearing blood debris, their release of reactive oxygen species (ROS), proteases, and neutrophil extracellular traps (NETs) can exacerbate blood–brain barrier disruption, promote neuroinflammation, and lead to microthrombosis (44, 46). Typically, neutrophil counts decline after about one week; persistent elevation indicates inadequate inflammation control and is strongly associated with DCI and poor prognosis (47, 48).

Monocytes similarly increase rapidly during early stages and migrate into brain tissue, differentiating into macrophages to phagocytose erythrocyte breakdown products (44). While aiding hematoma clearance, they also release pro-inflammatory mediators such as IL-6, TNF-α, and matrix metalloproteinases (MMPs), exacerbating brain injury and vasospasm (49). Entering the subacute phase, monocyte–macrophage lineages gradually shift toward the anti-inflammatory M2 phenotype, participating in tissue repair and regeneration. However, a renewed increase in monocytes during recovery may indicate complications such as impaired repair or hydrocephalus (50).

Comprehensive analysis using immune infiltration algorithms CIBERSORT and ssGSEA revealed significantly higher levels of activated neutrophils, activated dendritic cells, monocytes, and mast cells in the peripheral blood of aSAH patients compared to healthy controls, with notable differences in immune cell infiltration patterns between the two groups. Previous studies have demonstrated that neutrophil activation can promote ROS production (45, 51). Our findings also confirmed a link between ferroptosis and inflammation, further supporting the iron-inflammation axis as a potential therapeutic target for precise immunotherapy in aSAH (52, 53).

It is important to note that increased peripheral neutrophils and monocytes reflect systemic inflammation and iron-mediated oxidative stress but do not fully represent the immune microenvironment within the brain (54). Thus, while peripheral blood indicators are valuable for assessing systemic responses and screening prognostic biomarkers in aSAH, further research at the cerebrospinal fluid and brain tissue levels is required to clarify central-peripheral immune interactions and optimize intervention strategies.

The expanded mouse validation further supported the biological relevance of the four hub genes. Western blotting and immunofluorescence confirmed increased MAPK3/ERK1, STAT3, PGD, and G6PD protein expression in SAH mouse brain tissue, indicating that the transcriptomic changes were accompanied by protein-level alterations. In addition, increased MDA levels and reduced GPX4 activity indicated enhanced lipid peroxidation and impaired GPX4-dependent antioxidant defense after SAH. These biochemical findings provide additional support for ferroptosis-associated oxidative lipid injury in the SAH brain. Nevertheless, these data should be interpreted as evidence of ferroptosis-associated biochemical changes rather than definitive proof of ferroptotic cell death, because rescue experiments using ferroptosis inhibitors or gene-specific interventions were not performe.

The present molecular docking analysis prioritized Ganoderic Acid A (GAA) as an in silico compound predicted to interact with the four hub proteins. Previous studies in other neurological injury or ferroptosis-related models suggest that GAA may have antioxidant and anti-ferroptotic properties (55–61). However, the present study did not experimentally test GAA in aSAH models. Therefore, the docking results should be interpreted only as preliminary compound prioritization, not as evidence of therapeutic efficacy, direct binding, target engagement, or clinical translational potential in aSAH.

Several limitations warrant consideration. First, although this study integrated peripheral-blood transcriptomic analysis, public mouse cortex data, and single-cell RNA-seq data, the main discovery dataset was still derived from peripheral blood, and these datasets were not paired across the same individuals or species. Second, the four hub genes we prioritized, STAT3, MAPK3, G6PD, and PGD, are pleiotropic sensors of cellular stress, inflammation, and metabolism, so their up-regulation is unlikely to be specific to ferroptosis or to aSAH alone. Gene-specific loss- and gain-of-function studies are therefore needed to establish causality. Third, immune-cell infiltration was inferred solely from peripheral blood; this surrogate may not faithfully mirror the intracranial immune milieu after aneurysmal rupture. Future work should integrate paired cerebrospinal-fluid samples or peri-aneurysmal tissue to delineate central–peripheral immune crosstalk. Finally, although MDA and GPX4 activity assays supported ferroptosis-associated biochemical changes, definitive demonstration of ferroptotic cell death requires rescue experiments using ferroptosis inhibitors or gene-specific interventions. In addition, although data from other neurological-injury models suggest that GAA has anti-ferroptotic activity, its efficacy and safety in aSAH remain unexplored and will require dedicated pharmacokinetic and pharmacodynamic studies in aSAH-specific cellular and animal models.

## Conclusion

We identified MAPK3, STAT3, G6PD, and PGD as pivotal ferroptosis-associated hub genes in aSAH. These genes may constitute reliable biomarkers of ferroptotic activity in aSAH, while Ganoderic Acid A shows mechanistically grounded therapeutic potential to attenuate aSAH-related ferroptosis, thereby introducing a novel avenue for clinical intervention.

## Data Availability

The datasets analyzed in this study are publicly available in the Gene Expression Omnibus (GEO) https://www.ncbi.nlm.nih.gov/gds under accession numbers GSE36791, GSE79416, GSE16561, and GSE89866, and in the National Genomics Data Center (NGDC) https://ngdc.cncb.ac.cn/ under accession number OMIX006611.
