# Supplementary material for: Ferroptosis-associated candidate genes in aneurysmal subarachnoid hemorrhage: evidence from human peripheral blood and mouse brain
Source: Front Neurol. 2026 Oct 5;17:1823039. doi: 10.3389/fneur.2026.1823039 (PMC13677354; doi:10.3389/fneur.2026.1823039)
Supplement: Supplementary file 1 [file DataSheet_2.DOCX]

**s1 DEGs**

#################################################

library(limma)

#################################################

rm(list=ls())

options(stringsAsFactors = F)

#################################################

#################################################

design <- read.csv('design.csv',row.names = 1)

Expr <- read.csv('GSE36791.csv',row.names = 1)

################

########

library(limma)

contrast.matrix <- makeContrasts(Ruptured-Control , levels=design)

fit <- lmFit(Expr, design)

fit <- contrasts.fit(fit, contrast.matrix)

fit <- eBayes(fit)

output <- topTable(fit, coef=1,n=Inf,adjust.method='fdr')

sigGene <- output[which(output$P.Value<0.05 & abs(output$logFC) > log(1.2,2)),]

dim(sigGene)

upDEG<-output[which(output$P.Value<0.05 & output$logFC > log(1.2,2)),]

dim(upDEG)

dnDEG<-output[which(output$P.Value<0.05 & output$logFC < -log(1.2,2)),]

dim(dnDEG)

#################################################

#################################################

**S2 WGCNA**

rm(list=ls())

options(stringsAsFactors = F)

####################################

library(WGCNA)

library(limma)

library(tidyverse)

data <- read.csv('GSE36791.csv',row.names = 1)

design <- read.csv('design.csv',row.names = 1)

enableWGCNAThreads() #

powers = c(1:15) #

sft = pickSoftThreshold(datExpr0, powerVector = powers, verbose = 5)

pdf(file="3_independence.pdf",width=9,height=5)

par(mfrow = c(1,2))

cex1 = 0.9

######################################

plot(sft$fitIndices[,1], -sign(sft$fitIndices[,3])*sft$fitIndices[,2],

xlab="Soft Threshold (power)",ylab="Scale Free Topology Model Fit,signed R^2",type="n",

main = paste("Scale independence"));

text(sft$fitIndices[,1], -sign(sft$fitIndices[,3])*sft$fitIndices[,2],

labels=powers,cex=cex1,col="red");

abline(h=0.90,col="red") ##################################

plot(sft$fitIndices[,1], sft$fitIndices[,5],

xlab="Soft Threshold (power)",ylab="Mean Connectivity", type="n",

main = paste("Mean connectivity"))

text(sft$fitIndices[,1], sft$fitIndices[,5], labels=powers, cex=cex1,col="red")

dev.off()

#################

sft

softPower =sft$powerEstimate #

softPower

adjacency = adjacency(datExpr0, power = softPower)

TOM = TOMsimilarity(adjacency);

dissTOM = 1-TOM

geneTree = hclust(as.dist(dissTOM), method = "average");

pdf(file="4_gene.pdf",width=12,height=9)#

plot(geneTree, xlab="", sub="", main = "Gene clustering on TOM-based dissimilarity",

labels = FALSE, hang = 0.04)

dev.off()

minModuleSize = 25

dynamicMods = cutreeDynamic(dendro = geneTree, distM = dissTOM,

deepSplit = 2, pamRespectsDendro = FALSE,

minClusterSize = minModuleSize);

table(dynamicMods)#

dynamicColors = labels2colors(dynamicMods) #

table(dynamicColors) #

\# pdf(file="5_Tree.pdf",width=8,height=6)

plotDendroAndColors(geneTree, dynamicColors, "Dynamic Tree Cut",

dendroLabels = FALSE, hang = 0.03,

addGuide = TRUE, guideHang = 0.05,

main = "Gene dendrogram and module colors")

dev.off()

MEList = moduleEigengenes(datExpr0, colors = dynamicColors)

MEs = MEList$eigengenes

MEDiss = 1-cor(MEs);

METree = hclust(as.dist(MEDiss), method = "average")

pdf(file="6_module.pdf",width=7,height=6)

plot(METree, main = "Clustering of module eigengenes",

xlab = "", sub = "")

dev.off()

MEDissThres = 1.08

pdf(file="6_module_cut.pdf",width=7,height=6)

plot(METree, main = "Clustering of module eigengenes",

xlab = "", sub = "")

abline(h=MEDissThres, col = "red")

dev.off()

\###

nGenes = ncol(datExpr0)#

nSamples = nrow(datExpr0)#

moduleTraitCor = cor(MEs, datTraits, use = "p")#

moduleTraitPvalue = corPvalueStudent(moduleTraitCor, nSamples)#

pdf(file="8_Module.pdf",width=10,height=8)#

textMatrix = paste(signif(moduleTraitCor, 2), "\n(",

signif(moduleTraitPvalue, 1), ")", sep = "")

dim(textMatrix) = dim(moduleTraitCor)

par(mar = c(5, 10, 3, 3))

labeledHeatmap(Matrix = moduleTraitCor,

xLabels = names(datTraits),

yLabels = names(MEs),

ySymbols = names(MEs),

colorLabels = FALSE,

colors = greenWhiteRed(50),

textMatrix = textMatrix,

setStdMargins = FALSE,

cex.text = 0.5,

zlim = c(-1,1),

main = paste("Module-trait relationships"))

dev.off()

\###

moduleColors=dynamicColors

probes = colnames(datExpr0)#

geneInfo0 = data.frame(probes= probes,

moduleColor = moduleColors)

geneOrder =order(geneInfo0$moduleColor)#

geneInfo = geneInfo0[geneOrder, ]

\#

write.table(geneInfo, file = "all_genes.txt",sep="\t",row.names=F,quote=F)

for (mod in 1:nrow(table(moduleColors))){

modules = names(table(moduleColors))[mod]

probes = colnames(datExpr0)

inModule = (moduleColors == modules)

modGenes = probes[inModule]

write.table(modGenes, file =paste0("module_",modules,".txt"),sep="\t",row.names=F,col.names=F,quote=F)

}

modNames = substring(names(MEs), 3)

geneModuleMembership = as.data.frame(cor(datExpr0, MEs, use = "p"))

MMPvalue = as.data.frame(corPvalueStudent(as.matrix(geneModuleMembership), nSamples))

names(geneModuleMembership) = paste("MM", modNames, sep="")

names(MMPvalue) = paste("p.MM", modNames, sep="")

traitNames=names(datTraits)

geneTraitSignificance = as.data.frame(cor(datExpr0, datTraits, use = "p"))

GSPvalue = as.data.frame(corPvalueStudent(as.matrix(geneTraitSignificance), nSamples))

names(geneTraitSignificance) = paste("GS.", traitNames, sep="")

names(GSPvalue) = paste("p.GS.", traitNames, sep="")

picDir="module_trait"#

dir.create(picDir)

for (trait in traitNames){#

traitColumn=match(trait,traitNames)

for (module in modNames){

column = match(module, modNames)

moduleGenes = moduleColors==module

if (nrow(geneModuleMembership[moduleGenes,]) > 1){

pdfFile=paste("9_", trait, "_", module,".pdf",sep="")

outPdf=paste(picDir,pdfFile,sep="\\")

pdf(file=outPdf,width=7,height=7)

par(mfrow = c(1,1))

verboseScatterplot(abs(geneModuleMembership[moduleGenes, column]),

abs(geneTraitSignificance[moduleGenes, traitColumn]),

xlab = paste("Module Membership in", module, "module"),

ylab = paste("Gene significance for ",trait),

main = paste("Module membership vs. gene significance\n"),

cex.main = 1.2, cex.lab = 1.2, cex.axis = 1.2, col = module)

dev.off()

}

}

}

colnames(datExpr0)#

probes = colnames(datExpr0)

geneInfo0 = data.frame(probes= probes,

moduleColor = moduleColors)

for (Tra in 1:ncol(geneTraitSignificance))#

{

oldNames = names(geneInfo0)

geneInfo0 = data.frame(geneInfo0, geneTraitSignificance[,Tra],

GSPvalue[, Tra])

names(geneInfo0) = c(oldNames,names(geneTraitSignificance)[Tra],

names(GSPvalue)[Tra])

}

for (mod in 1:ncol(geneModuleMembership))#

{

oldNames = names(geneInfo0)

geneInfo0 = data.frame(geneInfo0, geneModuleMembership[,mod],

MMPvalue[, mod])

names(geneInfo0) = c(oldNames,names(geneModuleMembership)[mod],

names(MMPvalue)[mod])

}

geneOrder =order(geneInfo0$moduleColor)

geneInfo = geneInfo0[geneOrder, ]

write.table(geneInfo, file = "GS_MM.xls",sep="\t",row.names=F)#

########

nGenes = ncol(datExpr0)############

nSamples = nrow(datExpr0)############】

plotTOM = dissTOM^7

diag(plotTOM) = NA

pdf(file="10_allgenemap.pdf",width=9, height=9)############

TOMplot(plotTOM, geneTree, moduleColors, main = "Network heatmap plot, all genes")

dev.off()

################

nSelect = 400##############

set.seed(10)

select = sample(nGenes, size = nSelect)#########

selectTOM = dissTOM[select, select]

selectTree = hclust(as.dist(selectTOM), method = "average")

selectColors = moduleColors[select]#########

plotDiss = selectTOM^7

diag(plotDiss) = NA

pdf(file="11_selectgenemap.pdf",width=9, height=9)#########

TOMplot(plotDiss, selectTree, selectColors, main = "Network heatmap plot, selected genes")

dev.off()

############

pdf(file="12_dendrogram.pdf",width=8, height=8)

par(cex = 1.0)

plotEigengeneNetworks(MEs, "Eigengene dendrogram", marDendro = c(0,4,2,0), plotHeatmaps = FALSE)

dev.off()

##########

pdf(file="13_modulemap.pdf",width=8, height=8)

par(cex = 1.0)

plotEigengeneNetworks(MEs, "Eigengene adjacency heatmap", marHeatmap = c(3,4,2,2), plotDendrograms = FALSE, xLabelsAngle = 90)

dev.off()

##########

pdf(file="14_dendrogrammap.pdf", width=9, height=14)

par(cex = 0.9)

plotEigengeneNetworks(MEs, "", marDendro = c(0,4,1,2), marHeatmap = c(3,4,1,2), cex.lab = 0.8, xLabelsAngle= 90)

dev.off()

cytoDir="CytoscapeInput"##########

dir.create(cytoDir)

for (mod in 1:nrow(table(moduleColors))){ ##########

modules = names(table(moduleColors))[mod]

probes = colnames(datExpr0)

inModule = (moduleColors == modules)

modProbes = probes[inModule]

modGenes = modProbes

modTOM = TOM[inModule, inModule]

dimnames(modTOM) = list(modProbes, modProbes)

edges_File = paste("CytoscapeInput-edges-", modules , ".txt", sep="")

nodes_File = paste("CytoscapeInput-nodes-", modules, ".txt", sep="")

outEdge=paste(cytoDir,edges_File,sep="\\")

outNode=paste(cytoDir,nodes_File,sep="\\")

cyt = exportNetworkToCytoscape(modTOM,

edgeFile = outEdge,

nodeFile = outNode,

weighted = TRUE,

threshold = 0.02,

nodeNames = modProbes,

altNodeNames = modGenes,

nodeAttr = moduleColors[inModule])

}

##################################################################

**S3** CIBERSORT

rm(list=ls())

options(stringsAsFactors = F)

####################################

library('devtools')

library(CIBERSORT)

################

data("LM22")

result <- cibersort(sig_matrix=LM22,mixture_file=Expr,perm=1000,QN=T)

##########################################################################

##########################################################################

S4 ssGSEA

###################################

rm(list=ls())

options(stringsAsFactors = F)

###################################

library(GSVA)

#################

Expr_raw <- read.csv('GSE36791.csv',row.names = 1)

group <- read.csv('group.csv',row.names = 1)

IM27 <- read.csv('CellReports.csv',row.names =1,header = F)

sigGene <- read.csv('sigGene_Fc1.2.csv',row.names = 1)

###################################

#################

sigGene$gene_symbol <- row.names(sigGene)

gene_symbol <- sigGene$gene_symbol

Expr_raw <- normalizeBetweenArrays(Expr_raw)

Expr_raw <- t(Expr_raw)

Expr_1c <- Expr_raw[, colnames(Expr_raw) %in% gene_symbol]

geneSet <- t(IM27)

geneSet <- as.data.frame(geneSet)

######################

a <- geneSet

a <- a[1:nrow(a),]

set <- colnames(a)

l <- list()

for (i in set) {

x <- as.character(a[,i])

x <- x[nchar(x)!=0]

x <- as.character(x)

l[[i]] <-x

}

############################

geneSet2 <- l

Expr <- as.matrix(Expr_1c)

Expr <- t(Expr)

result <- gsva(Expr,geneSet2,method='ssgsea',kcdf='Gaussian',abs.ranking=TRUE,min.sz=1.1)

########################################################

############################
