# Supplementary material for: Ferroptosis-associated candidate genes in aneurysmal subarachnoid hemorrhage: evidence from human peripheral blood and mouse brain
Source: Front Neurol. 2026 Oct 5;17:1823039. doi: 10.3389/fneur.2026.1823039 (PMC13677354; doi:10.3389/fneur.2026.1823039)
Supplement: Supplementary file 3 [file Data_Sheet_3.DOCX]

Table S1

| Groups | aSAH (n = 10) | Non-SAH (n = 10) |
| --- | --- | --- |
| Age, years, mean ± SD | 60.2 (7.7) | 63.6 (8.9) |
| Sex (Male) | 5 | 4 |
| Hypertension (N) | 6 | 4 |
| Diabetes (N) | 1 | 3 |
| Smoking (N) | 5 | 6 |
| Drinking(N) | 3 | 5 |
| SAH Family history (N) | 0 | 0 |

Table S2: 35 Ferr-DEGs genes

| NO. | Ferr-DEGs | logFC | P.Value | adj.P.Val | Gene_status |
| --- | --- | --- | --- | --- | --- |
| 1 | PGD | 0.848320107 | 0.000000218 | 0.0000996 | up |
| 2 | SLC2A3 | 0.67597044 | 0.00003 | 0.00172743 | up |
| 3 | DUSP1 | 0.61106546 | 0.003332166 | 0.041187192 | up |
| 4 | SLC2A14 | 0.61033221 | 0.00015968 | 0.005072946 | up |
| 5 | SLC40A1 | 0.579585489 | 0.0000571 | 0.0025512 | up |
| 6 | LAMP2 | 0.578880216 | 0.00000252 | 0.000447162 | up |
| 7 | ACSL4 | 0.521896156 | 0.00295106 | 0.037912304 | up |
| 8 | CBS | 0.494685314 | 0.004834137 | 0.053570354 | up |
| 9 | SAT1 | 0.491475744 | 0.0000678 | 0.002827792 | up |
| 10 | NCF2 | 0.475912104 | 0.00000172 | 0.000361832 | up |
| 11 | SRXN1 | 0.47428745 | 0.0000482 | 0.002308027 | up |
| 12 | G6PD | 0.407178486 | 0.000410087 | 0.009573393 | up |
| 13 | MAP3K5 | 0.383021568 | 0.0000835 | 0.003270994 | up |
| 14 | ZFP36 | 0.380852599 | 0.002242101 | 0.031177643 | up |
| 15 | LPCAT3 | 0.37940127 | 0.001403199 | 0.022392291 | up |
| 16 | CHMP5 | 0.377904985 | 0.011666404 | 0.100830019 | up |
| 17 | STAT3 | 0.361207562 | 0.001059315 | 0.018305862 | up |
| 18 | TXNRD1 | 0.350921519 | 0.000673567 | 0.013517047 | up |
| 19 | JDP2 | 0.329693855 | 0.002777289 | 0.03645139 | up |
| 20 | HSD17B11 | 0.318195842 | 0.009776195 | 0.089100899 | up |
| 21 | MAPK3 | 0.317835789 | 0.000623693 | 0.012836359 | up |
| 22 | CAPG | 0.29244842 | 0.000275576 | 0.007305834 | up |
| 23 | NFE2L2 | 0.287970219 | 0.022382862 | 0.159239129 | up |
| 24 | DNAJB6 | 0.279031285 | 0.001825527 | 0.02707148 | up |
| 25 | FTH1 | 0.273162322 | 0.001950613 | 0.028334286 | up |
| 26 | ATG7 | 0.268296135 | 0.000780971 | 0.014871584 | up |
| 27 | ASNS | -0.279787958 | 0.001321143 | 0.021519581 | down |
| 28 | SLC2A6 | -0.280171813 | 0.043753535 | 0.248032287 | down |
| 29 | GOT1 | -0.316430808 | 0.000758614 | 0.014654256 | down |
| 30 | CS | -0.359324414 | 0.0000192 | 0.001320635 | down |
| 31 | AKR1C3 | -0.36324888 | 0.003692044 | 0.044235635 | down |
| 32 | LONP1 | -0.378858186 | 0.00099799 | 0.017571226 | down |
| 33 | XBP1 | -0.447978319 | 0.0000681 | 0.002836243 | down |
| 34 | RPL8 | -0.453724945 | 0.000456773 | 0.010321989 | down |
| 35 | PEBP1 | -0.738838537 | 6.79E-08 | 0.0000546 | down |

Table S3

| Primer No | Primer Name | Forward primer | Reverse primer |
| --- | --- | --- | --- |
| 1 | PGD | AGAAATTGAGACGGTGCCGA | CAGGGTTCTCCAGTTCCCAC |
| 2 | G6PD | TGACCTATGGCAACCGATACAA | CCGCAAAAGACATCCAGGAT |
| 3 | STAT3 | CAATACCATTGACCAGCCGAT | GAGCGACTCAAACTGCCCT |
| 4 | MAPK3 | CGGATCACAGTGGAGGAAGC | GACTGGCCCACCTCATCC |
| ref | GAPDH | CAAGGTCATCCATGACAACTTTG | GTCCACCACCCTGTTGCTGTAG |

Table S4：MCODE cluster scores and network characteristics

| **Cluster** | **Score** | **Nodes** | **Edges** |
| --- | --- | --- | --- |
| MCODE 1 | 4.286 | 8 | 15 |
| MCODE 2 | 4 | 4 | 6 |
| MCODE 3 | 3 | 3 | 3 |

Figure S1：Validation in Peripheral Blood Cells from Patients with Ischemic Stroke and traumatic brain injury

Figure S2：GSEA analysis of the top 4 hub genes.

(A-H) Represent the pathways about function enrichment of STAT3, MAPK3, G6PD, and PGD. GSEA, gene set enrichment analysis.
