# Supplementary material for: Ferroptosis-associated candidate genes in aneurysmal subarachnoid hemorrhage: evidence from human peripheral blood and mouse brain
Source: Front Neurol. 2026 Oct 5;17:1823039. doi: 10.3389/fneur.2026.1823039 (PMC13677354; doi:10.3389/fneur.2026.1823039)
Supplement: Supplementary file 4 [file Data_Sheet_1.DOCX]

STAT3

88kda

PGD

53kDa

G6PD

59kDa

β-actin

42kDa
